# The genetic landscape of anaplastic astrocytoma

**DOI:** 10.18632/oncotarget.1505

**Published:** 2013-10-16

**Authors:** Patrick J. Killela, Christopher J. Pirozzi, Zachary J. Reitman, Sian Jones, B. Ahmed Rasheed, Eric Lipp, Henry Friedman, Allan H. Friedman, Yiping He, Roger E. McLendon, Darell D. Bigner, Hai Yan

**Affiliations:** ^1^ Department of Pathology, Duke University Medical Center, The Preston Robert Tisch Brain Tumor Center at Duke, and Pediatric Brain Tumor Foundation Institute at Duke, Durham, NC; ^2^ Personal Genome Diagnostics, Inc. 2809 Boston St, Suite 503, Baltimore, MD

**Keywords:** Exome sequencing, Anaplastic Astrocytoma, IDH1, Notch

## Abstract

Anaplastic astrocytoma WHO grade III (A3) is a lethal brain tumor that often occurs in middle aged patients. Clinically, it is challenging to distinguish A3 from glioblastoma multiforme (GBM) WHO grade IV. To reveal the genetic landscape of this tumor type, we sequenced the exome of a cohort of A3s (n=16). For comparison and to illuminate the genomic landscape of other glioma subtypes, we also included in our study diffuse astrocytoma WHO grade II (A2, n=7), oligoastrocytoma WHO grade II (OA2, n=2), anaplastic oligoastrocytoma WHO grade III (OA3, n=4), and GBM (n=28). Exome sequencing of A3s identified frequent mutations in IDH1 (75%, 12/16), ATRX (63%, 10/16), and TP53 (82%, 13/16). In contrast, the majority of GBMs (75%, 21/28) did not contain IDH1 or ATRX mutations, and displayed a distinct spectrum of mutations. Finally, our study also identified novel genes that were not previously linked to this tumor type. In particular, we found mutations in Notch pathway genes (NOTCH1, NOTCH2, NOTCH4, NOTCH2NL), including a recurrent NOTCH1-A465Tmutation, in 31% (5/16) of A3s. This study suggests genetic signatures will be useful for the classification of gliomas.

## INTRODUCTION

Gliomas are the most common primary tumor of the central nervous system and are classified from Grade I to Grade IV on the basis of histopathological and clinical criteria established by the World Health Organization (WHO) [1, 2]. The Grade II tumors, which include diffuse astrocytomas (A2) and well-differentiated oligodendrogliomas (O2), are slow growing and tend to progress into Grade III tumors. The grade III tumors include anaplastic astrocytomas (A3) and anaplastic oligodendrogliomas (O3), and are faster growing and more aggressive. These tumors often invade neighboring tissue and are able to progress into Grade IV secondary glioblastoma multiforme (GBM). Primary GBM is a genetically and clinically unique disease which arises *de novo*. GBMs are the most lethal form of gliomas and have the propensity to infiltrate normal surrounding tissue, making complete resection difficult to accomplish thus resulting in a poor prognosis. In clinics, it is often very difficult to distinguish a GBM from a contrast-enhancing A3 lesion by magnetic resonance imaging or histopathology alone. In addition to these purely astrocytic tumors, there are also Grade II and Grade III oligoastrocytomas which present with histocytological appearances of oligodendrogliomas and astrocytomas, which can also progress to GBMs. These “mixed histology” tumors present diagnostic challenges and their classification often varies between institutions [1, 3].

The fatal nature of GBMs together with the availability of only a few minimally efficacious FDA approved treatment modalities led to the ambitious undertaking of sequencing the GBM genome in the hopes of finding unique genetic alterations to help classify tumors and identify potential therapeutic targets [4-8]. While this pursuit has proved fruitful in identifying several key genes involved in GBM tumorigenesis, relatively little has been done for the genome wide sequencing of progressive astrocytomas, including A2s, A3s, and secondary GBMs. In an effort to establish the genetic landscape of progressive astrocytomas, we have sequenced the exome of a total of 57 gliomas, 30 of which were progressive astrocytomas or oligoastrocytomas (A2, n=7; A3, n=16; OA2, n = 2; OA3, n = 4; secondary GBM, n=1) and 27 of which were primary GBMs. Our study revealed that mutations in *IDH1*, *ATRX*, and *TP53* are the most frequent genetic alterations in progressive astrocytomas. Novel alterations, including recurrent mutations in *PIK3R1* and Notch family genes (*NOTCH1, NOTCH2, NOTCH4, NOTCH2NL*) are also revealed in the *IDH1*-mutated astrocytomas. Additionally, our data further supports that on a genetic level, primary GBMs displayed distinct mutation spectrums differing from those of progressive astrocytomas.

## RESULTS

### Exome sequencing of astrocytic tumors

To establish the genetic landscape of progressive astrocytomas, we sequenced matched tumor and normal pairs for 57 progressive astrocytomas and GBM exomes from DNA samples collected at the Preston Robert Tisch Brain Tumor Center at Duke University (Supplementary Table 1). Utilizing the Agilent SureSelect Exome platform, libraries on average yielded 18.2G bases with 94.5% of targeted regions represented by at least 10 high quality reads (Supplementary Table 2). Our sample cohort including 23 A2s and A3s, 6 OA2s and OA3s, and 28 GBMs identified 2,003 somatic mutations and 645 copy number alterations (Supplementary Table 3 and 4). To assess the accuracy of our mutation calling criteria, 255 mutations were selected for verification, accounting for all genes that were mutated in 3 or more tumors. 92% of these mutations could be successfully amplified via Sanger sequencing, of these, 95% were verified (Supplementary Table 5).

### Genetic alterations identified in A2s

**Figure 1:**
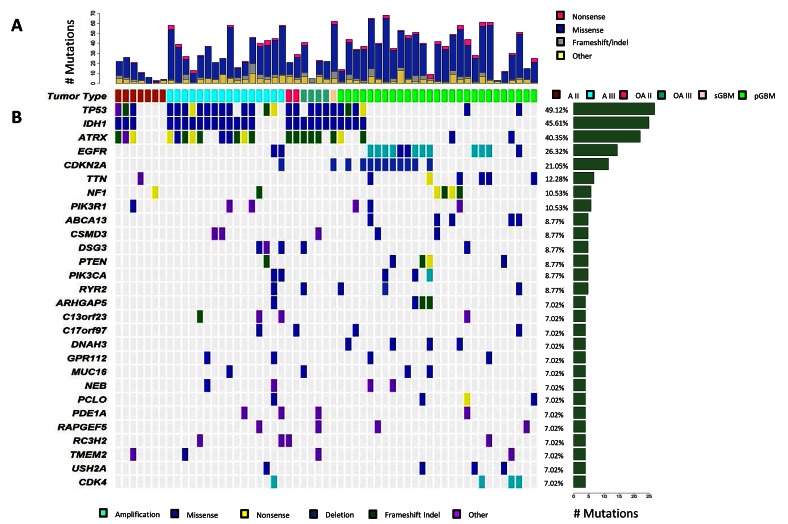
Genetic landscape of progressive astrocytomas Mutational analysis utilizing exome sequencing of matched tumor and normal pairs for 57 progressive astrocytomas, oligoastrocytomas, and GBMs. (A) Distribution of mutation types (Nonsense, Missense, Frameshift/Indel, Other) are reported for each tumor. (B) Depiction of Mutation Spectrum for each tumor is shown. Represented are genes which were mutated in four or more gliomas. The frequency (%) and number of gene alterations in the tumor cohort is represented on the right.

Exome sequencing of seven A2s revealed 93 somatic mutations. On average, A2s contained 13 somatic mutations, with 92% of targeted regions covered by 10x high quality reads or more. *IDH1*, *ATRX*, and *TP53* were co-mutated in 3/7 (43%) of our patient cohort, and no other gene was mutated in more than one grade II astrocytoma (Figure 1). We did not find any high copy number gain or deletion by our methodology in A2s.

### Genetic alterations identified in A3s

Exome sequencing of 16 A3s revealed 576 mutations. On average, A3s contained 36 somatic mutations with 92% of targeted regions covered by 10x high quality reads or more. Mutations in the genes *IDH1*, *ATRX*, and *TP53* were the most frequent events in A3s confirming previously published studies identifying them as critical astrocytoma derived mutations (Figure 1) [9-12]. Copy number alterations were infrequent in A3s, only 3 of 16 tumors contained detectable alterations via exome analysis with a median of 22 genes targeted by copy number alterations (range: 2-47) in our cohort (Supplementary Table 4).

Notch signaling pathway disruption has been previously reported in low grade gliomas [13, 14]. Here, we report mutations in Notch pathway members in 5/16 (31%) of A3s. *NOTCH1, NOTCH2, NOTCH4,* and *NOTCH2NL* were mutated in 2, 1, 1, and 1 cases, respectively. Notably, we observed a recurrent *NOTCH1* missense A465T mutation in 2 cases. This mutation resides within the extracellular epidermal growth factor-like (EGF) repeats of the Notch1 protein [15, 16]. We also observed two *PIK3CA* and two *PIK3R1* mutations in 16 A3 tumors. Sequencing also revealed novel recurrent mutation in *desmoglien 3* (*DSG3*), a calcium binding transmembrane glycoprotein present in desmosomes [17], in 3/16 (19%) of A3s, in 1/4 OA3s (25%), and in 1/28 (4%) GBMs. All five *DSG3* alterations occurred in *IDH1* wild type tumors.

Of great interest to the brain tumor community is the evolution of genetic mutations as astrocytomas progress to higher grade lesions. To this end we have performed exome sequencing on a pair of astrocytomas that progressed from A2 (Tumor P110) to an A3 (Tumor P112). While both tumors harbored the same number of mutations, the mutational spectrum was quite distinct. Two genes, *IDH1* and *ADRBK1*, contained mutations at the exact same residue in both tumors. Furthermore, both P110 and P112 contained *TP53* mutations. However, the mutation was located at a different residue within TP53, R273C in P110 and P177R in P112. These results suggest that mutations in *IDH1* are an early gene mutation, and that progressive tumors result from independent clonal expansions from a common IDH1-mutated population of cells.

### Genetic alterations identified in OAs

OAs present a great diagnostic challenge to neuropathologists as they show histological properties of both astrocytomas and oligodendrogliomas [1]. Recently, studies have suggested that at least genetically, the majority of these “mixed histology” tumors contain genetic events representative of either astrocytomas, namely mutations in *IDH1*, *ATRX*, and *TP53*, or oligodendrogliomas, namely chr 1p/19q LOH and mutations in *CIC* and/or *FUBP1* [9, 11, 18, 19]. We assessed exomes of two OA2s and four OA3s, revealing 157 somatic mutations. On average OAs contained 26 somatic mutations (range: 21-41), and 94% of targeted regions were covered by 10x or more high quality reads. *IDH1* (100%, 6/6), *ATRX* (83%, 5/6), *and TP53* (83%, 5/6) were the most commonly mutated genes in this cohort (Figure 1).

### Genetic alterations identified in GBMs

To compare the genetic landscape of A2, A3, OA2, OA3 and secondary GBMs to primary GBMs, we next sequenced the exome of 28 GBMs. Exome sequencing identified 1,177 somatic mutations. Primary GBMs on average contained 42 somatic mutations with 93% of targeted regions covered by 10x high quality reads or more. Copy number alterations were frequent in primary GBMs, averaging 32 events per tumor (range: 1-132) (Supplementary Table 4).

Confirming previous studies, the EGFR/PTEN/PI(3)K pathway is the most frequently affected pathway in GBMs [4, 5, 8, 20, 21]. We found frequent genetic alterations of *EGFR* in 13/28 (46%), *PTEN* mutations in 4/28 (14%), *PIK3CA* mutations in 3/28 (10%), and *PIK3R1* mutations in 3/28 (10%) of the GBMs (Fig. 1). However, *EGFR*, *PIK3CA* and *PIK3R1* were also mutated in lower grade tumors (Fig. 1). We found two *EGFR* (13%), two *PIK3CA* (13%), two *PIK3R1* (13%), and one *PTEN* (7%) mutation from A3s, and one *PIK3R1* mutation from A2s. *PTEN*, *PIK3CA* and *PIK3R1* mutations were mutually exclusive. Furthermore, *PIK3CA* mutations were found exclusively in *IDH1-*wildtype tumors (*P*=.05, Fisher's exact test, two-tailed) whereas four of six *PIK3R1* mutations were found in the *IDH1* mutant subgroup (*P*=0.39, Fisher's exact test, two-tailed). Within our GBM cohort were five GBMs harboring mutations in *IDH1* and/or *ATRX*, mutations typically associated with progressive astrocytomas.

## DISCUSSION

Despite decades of research, the prognosis for patients with malignant gliomas remains dismal. Recently, significant progress has been made in elucidating the genetic aberrations in GBMs [4-8, 21, 22]. We sought to determine the genetic landscape of A3s and compare the mutation spectrum to other subtypes of gliomas including A2s, OAs, and GBMs. Our results represent the largest scale of exomic sequencing of progressive astrocytomas to date. Here, we report that mutations in *IDH1*, *ATRX*, and *TP53* are particularly prevalent in A3s; whereas *EGFR* and *CDKN2A* were the most frequently altered genes in GBMs, a finding that corroborates previous studies [3, 9, 10, 23]. These findings will aid in improving the classification of brain tumors, and the selection of patients with genetically homogeneous tumors for clinical trials.

Several genes not previously linked to gliomas were identified in this study. We found *DSG3* mutated in 19% of A3s, all of which do not harbor the *IDH1* mutation. *DSG3* has been reported to be expressed at high levels in head and neck squamous cell carcinoma, and has been implicated as a potential biomarker for detection of this cancer's lymph node metastases [24]. Furthermore, within A3s we identified frequent mutations (5/16, 31%) in members of the Notch pathway (*NOTCH1*, *NOTCH2*, *NOTCH4*, *NOTCH2NL*). Notch family members have been reported as differentially expressed in astrocytomas and have been implicated in gliomagenesis [14, 25]. We observed a recurrent missense mutation among two astrocytomas converting amino acid 465, alanine to threonine. NOTCH1-A465T is located within an EGF like repeat domain, where additions of O-fucose to Ser/Thr is predicted and resides near a critical GlcNAc'ylation site [26, 27]. This exact residue is also reported mutated in one colon adenocarcinoma in the COSMIC database (Sample ID COS1863429), suggesting this may be a hotspot mutation that may play a role in other cancer types. The spectra and frequency of Notch mutations we observed in astrocytomas further supports the notion of Notch pathway aberrations as a critical player in astrocytoma transformation.

Our exome sequencing of primary GBMs confirmed previous findings, highlighting frequent mutations in *EGFR* (46%), deletions of *CDKN2A* (39%), *TP53* mutations (25%), *NF1* mutations (15%), and *PTEN* mutations (15%) in primary GBMs [4, 5, 8]. Recent reports have identified *TET2*, a gene encoding the enzyme which catalyzes 5-methylcytosine to 5-hydroxymethylcytosine, to be frequently mutated in AML, and TET mutations are mutually exclusive with *IDH1/2* mutations in AML [28-30]. Mutations in *IDH1/2* or *TET* have resulted in epigenetic alterations including a hypermethylated phenotype in gliomas and AML, respectively [28, 31]. It is of interest to note that we observed two *TET2* mutations in our GBM subset and that one primary GBM, P134, harbors a *TET2* mutation and an *IDH1* R132 mutation. Additional investigation of the epigenetic features of this tumor is necessary to make further conclusions about the potential synergy between these two epigenetic modifiers. Overall, the data contained here represents the largest exome sequencing study of progressive gliomas to date. We have elucidated the genetic landscape of progressive gliomas encompassing A2s, OA2s, A3s, OA3s, secondary GBMs, and primary GBMs, uncovering genes not previously linked to progressive astrocytomas. Furthermore, this study highlights the vast genetic differences between progressive astrocytomas and primary GBMs, providing further evidence of two uniquely distinct disease entities.

## METHODS

### Sample Collection and Processing

Tumor samples and corresponding clinical information were obtained with consent and Institutional Review Board approval from the Preston Robert Tisch Brain Tumor Center BioRepository at Duke University in accordance with the Health insurance Portability and Accountability Act. Tissue sections were reviewed by board certified neuropathologists to confirm diagnosis and to ensure sections contain ≥ 95% tumor cells. DNA was extracted from snap frozen tumors and normal blood in 16 grade III astrocytomas, 7 grade II astrocytomas, 2 grade II Oligoastrocytomas, 4 grade III Oligoastrocytomas, and 28 glioblastomas and processed for exome sequencing. Secondary GBM designates tumors which were resected > 1 year after a prior diagnosis of a low grade glioma (Grade II-III).

### Methods for Cancer Genome Analysis

Genomic purification, library construction, exome capture, next generation sequencing, and bioinformatic analyses of tumor and normal samples were performed at Personal Genome Diagnostics (Baltimore, MD). In brief, genomic DNA from tumor and normal samples were fragmented and used for Illumina TruSeq library construction (Illumina, San Diego, CA). Exonic regions were captured in solution using the Agilent SureSelect 51 Mb kit (version 4) according to the manufacturer's instructions (Agilent, Santa Clara, CA). Paired-end sequencing, resulting in 100 bases from each end of the fragments, was performed using a HiSeq 2000 Genome Analyzer (Illumina, San Diego, CA). The tags were aligned to the human genome reference sequence (hg18) using the Eland algorithm of CASAVA 1.7 software (Illumina, San Diego, CA). The chastity filter of the BaseCall software of Illumina was used to select sequence reads for subsequent analysis. The ELAND algorithm of CASAVA 1.7 software (Illumina, San Diego, CA) was then applied to identify point mutations and small insertions and deletions. Known polymorphisms recorded in dbSNP were removed from the analysis. Potential somatic mutations were filtered and visually inspected as described previously [32].

### Mutation Validation

Genes which contain mutations in 3 or more tumors were selections for mutational validation utilizing Sanger sequencing technologies as described previously [33], accounting for 255 mutations (Supplementary Table 5). All PCR Primers were designed using Primer3 to generate PCR products of 300-500 bases.

## SUPPLEMENTARY TABLES


